# The Association Between the Weight-Adjusted Waist Index and Dementia Risk in Older Adults: A Prospective Cohort Study

**DOI:** 10.3390/healthcare13111286

**Published:** 2025-05-29

**Authors:** Xiaowen Suo, Yiming Wang, Fuzhong Xue

**Affiliations:** 1 Department of Biostatistics, School of Public Health, Cheeloo College of Medicine, Shandong University, Wenhuaxi Road, P.O. Box 10044, Jinan 250012, China; 202016361@mail.sdu.edu.cn; 2 Department of Biotechnology, College of Life Sciences, Shandong Agricultural University, Taian 271018, China; 202434613@mail.sdu.edu.cn

**Keywords:** cohort study, dementia, older adults, United Kingdom Biobank, weight-adjusted waist index

## Abstract

**Background/Objectives:** The weight-adjusted waist index (WWI), a novel measure of abdominal obesity independent of overall weight, has shown promise in assessing health risks. However, its relationship with dementia remains unclear. This study investigates the association between the WWI and dementia risk in British older adults. **Methods**: Employing data from the United Kingdom Biobank, we conducted a prospective cohort study focusing on 155,683 elderly participants. Multivariable logistic regression models were employed to investigate the linear association between the WWI and all-cause dementia. Restricted cubic spline analysis was used to evaluate non-linear relationships, while subgroup analyses and interaction tests were performed to examine the stability of findings across different populations. **Results**: Among participants (mean age: 63.2 years; 52.2% male), 3211 dementia cases were identified during 13.1 years of follow-up (2.06% prevalence). The analysis revealed a significant inverse relationship between the WWI and dementia risk. In fully adjusted models, higher WWI was associated with a reduced risk of dementia (β = −0.274, *p* < 0.001). When the WWI was categorized into quartiles, participants in the highest quartile exhibited a 47% lower risk of dementia compared to those in the lowest quartile. This inverse association was particularly pronounced among non-current smokers (*p* < 0.05 for interaction). Restricted cubic spline analysis confirmed a non-linear relationship, with the protective effect of the WWI becoming more evident at higher values. **Conclusions**: The WWI is inversely associated with dementia risk in British older adults. These findings reveal that the WWI may serve as a potential predictor for dementia risk, highlighting its potential in risk stratification and prevention strategies.

## 1. Introduction

Dementia is a neurodegenerative disorder marked by the progressive deterioration of cognitive functions, resulting from pathological changes that damage neurons and brain tissues [1]. This cognitive decline extends beyond the natural aging process, causing substantial impairments in memory, reasoning, and daily functioning [2,3]. Dementia is a global health challenge, impacting more than 55 million individuals worldwide. With approximately 10 million new cases diagnosed annually, it ranks as the seventh leading cause of mortality and represents a significant driver of disability and dependency in aging populations across the globe [4]. In 2019, the global economic impact of dementia was estimated to be USD 1.3 trillion, with informal caregivers, including family members and close friends, accounting for nearly half of these costs through their provision of daily care and support [5]. Given this substantial impact, identifying modifiable risk factors is essential for decreasing the occurrence and prevalence of dementia.

The association between obesity and dementia has been widely studied, yet the findings are mixed [6,7,8]. Some studies report a positive correlation between obesity and an elevated risk of dementia [9,10,11,12,13,14], while others indicate a potential protective effect [15,16,17,18,19,20]. These divergent results underscore the complexity of the obesity–dementia relationship and the need for further research to clarify its underlying mechanisms. Conventionally, body mass index (BMI) has been widely utilized as a key measure for evaluating obesity in a multitude of studies [21]. However, the shortcomings of BMI as a holistic indicator of obesity have become more apparent, especially its failure to precisely capture the distribution of body fat [22,23]. Recently, the weight-adjusted waist index (WWI) has emerged as a promising new anthropometric measure that provides a comprehensive body composition assessment by integrating both fat and muscle mass [24,25]. It standardizes waist circumference (WC) based on body weight, enhancing the practicality of WC while reducing its association with BMI. Moreover, the WWI overcomes the “obesity paradox” often seen with BMI, where the relationship between obesity and health outcomes is hindered by BMI’s failure to account for variations in body fat distribution, particularly in aging populations [26]. By adjusting waist circumference in relation to body weight, the WWI offers a more accurate and consistent assessment of body composition and its associated health outcomes.

As a new indicator for evaluating abdominal obesity that is independent of overall body weight, the WWI has demonstrated potential in assessing health risks. Cao et al. identified a U-shaped relationship between the WWI and all-cause mortality, indicating that the WWI is a superior predictor of all-cause mortality compared with BMI and WC [27]. Li et al. demonstrated that the WWI outperforms other obesity indicators (BMI, waist-to-height ratio, WC, height, and weight) in predicting chronic kidney disease and albuminuria. Li et al. discovered that higher WWI levels are linked to a greater chance of experiencing depression [28]. Sun et al. indicated that there is a notable connection between increased WWI levels and the likelihood of developing hypertension in the elderly population [29]. Zheng et al. revealed that WWI is positively correlated with the risk of type 2 diabetes mellitus in adults within the United States [30]. Zhang et al. established a linear and substantial relationship between the WWI and the prevalence of heart failure in a general population sample [31]. Research conducted by Lin et al. indicated that in the case of elderly Chinese men, a greater WWI value was associated with declined cognitive performance [32]. Zhao et al. found that elevated levels of the WWI are linked to diminished cognitive abilities in Americans aged 60 and older [33]. When it comes to dementia, research on the WWI and dementia is limited to two cross-sectional studies conducted in Chinese populations. Wang et al. investigated the distinct associations of multiple adiposity indices, such as the WWI, with dementia among rural Chinese older adults, revealing a linear correlation between the WWI and dementia risk [34]. A positive and independent relationship between the WWI and dementia was identified by Zhou et al. in their study of Chinese individuals with hypertension [35]. The applicability of these findings to other populations remains unknown.

To our knowledge, existing studies on the WWI–dementia association have been exclusively conducted in Chinese populations, with no evidence available for other ethnic groups. Our study fills this critical gap by investigating the relationship between the WWI and dementia in British aging populations, providing novel perspectives on the potential of the WWI as a novel indicator for dementia in diverse ethnic and geographic populations.

## 2. Materials and Methods

### 2.1. Study Population

This cohort study aimed to investigate the association between the WWI and dementia in older adults using data from the United Kingdom (UK) Biobank. The UK Biobank is a comprehensive biomedical data repository that has played a significant role in advancing scientific understanding in various fields related to health and disease. It has collected extensive data, including demographics, lifestyle, clinical measurements, and genetic data, from over 500,000 adults living in England, Scotland, or Wales [36]. Data collection at baseline took place between 2006 and 2010.

**Figure 1:**
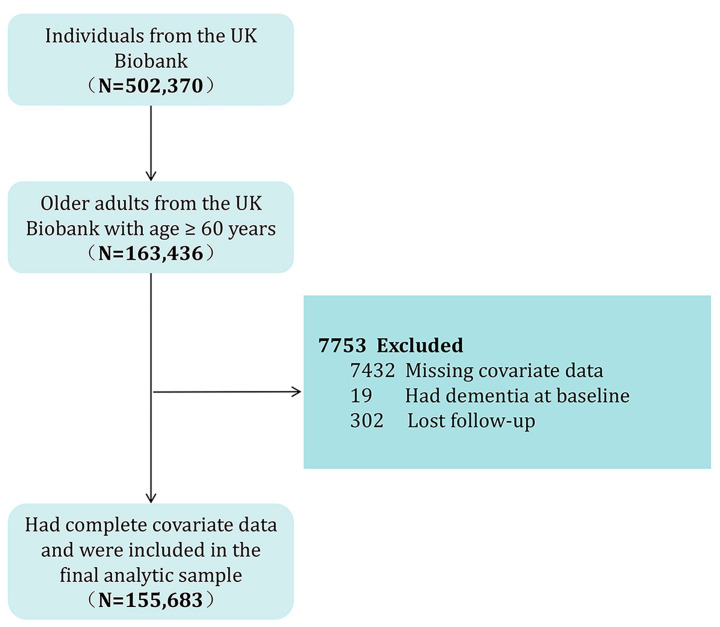
Flowchart of participant selection.

The exclusion criteria for participants in this analysis included the following: (1) had dementia at the time of participation; (2) experienced loss to follow-up during the study period; (3) age < 60 years old; and (4) incomplete covariate data. After applying the exclusion criteria, all remaining 155,683 participants with complete data were included in the final analysis cohort. Figure 1 illustrates a flowchart detailing the participant selection process.

### 2.2. Assessment of Dementia

All-cause dementia was used as the outcome variable in this study. All-cause dementia is a clinical syndrome characterized by progressive cognitive decline (e.g., memory, reasoning, or language impairments) severe enough to interfere with daily functioning, resulting from multiple underlying etiologies (e.g., Alzheimer’s disease, vascular dementia, Lewy body dementia). Dementia diagnosis and classification followed the International Statistical Classification of Diseases, Tenth Revision (ICD-10) codes. Cases of all-cause dementia were identified using the ICD-10 codes A81.0, F00, F01, F02, F03, F05.1, F10.6, G31.0, G31.1, G31.8, G30, and I67.3. These codes encompass various dementia subtypes, including Alzheimer’s disease, vascular dementia, Lewy body dementia, unspecified dementia, and other specified subtypes. Dementia outcomes were identified using predefined algorithms (Category 47) and first occurrences (Category 1712) within the UK Biobank datasets, with subsequent validation conducted by the UK Biobank outcome adjudication team [37]. Case identification relied on linked data from primary care, hospital inpatient records, and death registers. The follow-up period extended from the recruitment date (March 2006) until the occurrence of the first dementia diagnosis, death, or the conclusion of the study period (December 2022), depending on whichever event occurred earliest.

### 2.3. Assessment of Weight-Adjusted Waist Index

WWI is a novel anthropometric indicator that standardizes WC relative to body weight [24]. In this study, the WWI served as the primary exposure variable. It was calculated by dividing WC (in centimeters) by the square root of body weight (in kilograms) [24]. Weight and WC data were collected during the initial assessment phase of the UK Biobank study. The WWI was analyzed both continuously and categorically, with categories defined by quartiles.

### 2.4. Assessment of Covariates

Based on established research on dementia risk factors [38], this study identified and adjusted for eight covariates: age, sex, ethnicity, BMI, education level, alcohol consumption, smoking status, and chronic conditions. BMI was obtained by dividing weight in kilograms by height in meters squared. Alcohol consumption was divided into never drinkers and ever drinkers. Smoking status was categorized as either current smokers or non-current smokers, with the latter group including those who never smoked and former smokers. Educational attainment was grouped into two levels: those with a college or university degree and those without. Ethnicity was self-reported using a structured questionnaire with predefined options and was classified into five groups: White, Mixed, Asian, Black, or Other. Chronic conditions were determined based on physician-diagnosed diseases, including cardiovascular or heart-related issues (such as heart attack, angina, stroke, or hypertension), diabetes, or cancer.

### 2.5. Statistical Analysis

The association between the WWI and dementia was evaluated using multivariable logistic regression models across three models, with outcomes expressed as odds ratios (ORs) and 95% confidence intervals (CIs). Model 1 included no covariate adjustments; Model 2 adjusted for age, sex, and ethnicity; and Model 3 further incorporated adjustments for age, sex, BMI, alcohol consumption, smoking status, education level, ethnicity, and chronic conditions. To enhance sensitivity, the WWI was transformed from a continuous variable into quartiles. To examine potential non-linear relationships between the WWI and dementia, restricted cubic splines with five knots were utilized. Additionally, subgroup analyses were performed to assess the relationship across various demographic and lifestyle factors, including sex, smoking status, alcohol use, educational attainment, ethnicity, and chronic health conditions. To assess potential heterogeneity in the association, interaction tests were carried out to ascertain whether the relationship varied significantly among predefined subgroups. We defined statistical significance as *p* < 0.05. The analyses were implemented in R software (Version 4.4.1).

## 3. Results

### 3.1. Baseline Characteristics

A total of 155,683 older individuals (mean age = 63.23 ± 2.36 years) were included in the study, with 52.2% (n = 81,314) being male and 47.8% (n = 74,369) female. During a median follow-up period of 13.1 years, 3211 cases of dementia were recorded, indicating a prevalence rate of 2.06% within the study population. The average BMI among participants was recorded at 27.57 kg/m^2^, while the mean WWI for the overall group was 8.05 cm/√kg. Comprehensive details regarding the participants’ characteristics can be found in Table 1. The study population was stratified into quartiles of the WWI, defined as Q1 (<7.31 cm/√kg), Q2 (7.31–7.99 cm/√kg), Q3 (7.99–8.72 cm/√kg), and Q4 (>8.72 cm/√kg). Dementia prevalence decreased with increasing WWI quartiles. In contrast to individuals from the lowest WWI quartile, people in the highest quartile presented a higher prevalence of female gender, non-smoking, and alcohol consumption history. Moreover, they were more likely to have had a previous heart attack, stroke, angina, or hypertension. Additionally, individuals with elevated WWI levels tended to exhibit increased BMI values.

### 3.2. Association Between WWI and Dementia

**Figure 2:**
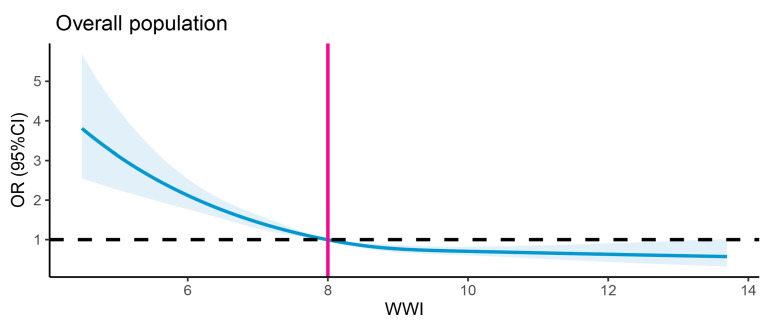
Restricted cubic spline analysis of association between WWI and risk of all-cause dementia. The black dashed line denotes the reference line (OR = 1), while the vertical gray line indicates the value of WWI corresponding to OR = 1. The solid blue curve indicates the OR estimates.

**Figure 3:**
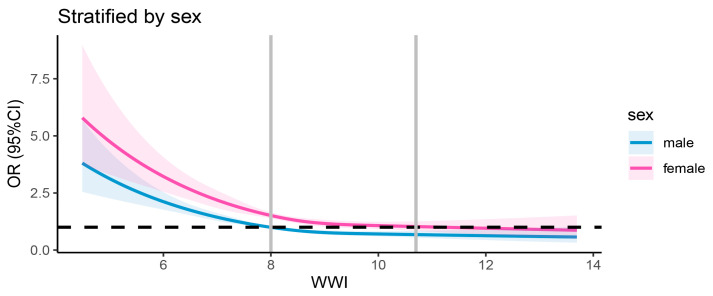
Sex-stratified restricted cubic spline analysis of association between WWI and all-cause dementia risk. The black dashed line denotes the reference line (OR = 1), while the vertical gray line indicates the value of WWI corresponding to OR = 1. The solid blue curve represents the OR estimates for males, and the solid pink curve represents the OR estimates for females.

The results regarding the association between the WWI and dementia are detailed in Table 2. Multivariable logistic regression analyses revealed consistent negative associations between the WWI (treated as a continuous variable) and dementia across three models. The β coefficient was −0.047 in the first model, −0.085 in the second model, and −0.274 in the third model. When WWI was categorized into quartiles, the inverse association with dementia remained statistically significant across all quartiles. In Model 1, participants in Quartile 2 (7.31–7.99 cm/√kg) demonstrated a 9% lower risk of dementia (0.91, 0.83–1.01), which further decreased to 15% in Model 2 (0.85, 0.77–0.94) and 23% in Model 3 (0.77, 0.69–0.85). For Quartile 3 (7.99–8.72 cm/√kg), the risk reduction was 15% in Model 1 (0.85, 0.76–0.92), 25% in Model 2 (0.75, 0.67–0.83), and 39% in Model 3 (0.61, 0.54–0.69). The most significant reduction was observed in Quartile 4 (>8.72 cm/√kg), with a 13% lower risk in Model 1 (0.87, 0.79–0.96), 23% in Model 2 (0.77, 0.69–0.85), and 47% in Model 3 (0.53, 0.46–0.62). When fully adjusted, the β values for the second, third, and fourth WWI quartiles were −0.26, −0.49, and −0.63, respectively, relative to the first quartile. The trend tests further supported these findings, showing a statistically significant dose–response relationship between increasing WWI quartiles and decreasing dementia risk. The *p*-values for trend were highly significant across all models (all *p* for trend < 0.0001), suggesting a consistent inverse association between WWI and dementia risk. Notably, the dose–response relationship between the WWI and dementia incidence demonstrated a non-linear pattern (*p* < 0.0001 for non-linearity). The association pattern was similar between sexes. Figure 2 displays the restricted cubic spline analysis illustrating the association between the WWI and the risk of all-cause dementia. Figure 3 presents the sex-stratified restricted cubic spline analysis depicting the association between the WWI and the risk of all-cause dementia.

### 3.3. Subgroup Analysis

To probe into potential variations among diverse population subgroups, subgroup analyses and interaction tests were carried out. The stratification was based on sex, ethnicity, education level, alcohol consumption, smoking status, and chronic conditions. Table 3 presents the results of the subgroup analysis examining the association between the WWI and dementia. The inverse association between the WWI and dementia remained consistent across all subgroups, including those categorized by sex, age, ethnicity, education level, and chronic conditions. Notably, as presented in Table 3, a significantly stronger inverse relationship between the WWI and dementia was observed among non-current smokers (*p* < 0.05).

## 4. Discussion

This prospective cohort study, comprising 155,683 older adults with data from the UK Biobank, revealed significant inverse associations between the WWI and incident dementia risk. Specifically, those with reduced WWI levels were associated with an elevated likelihood of dementia relative to individuals with higher WWI values. This association was consistently observed across different demographic groups, as evidenced by subgroup analyses and interaction tests. These groups included sex, smoking status, alcohol intake, education level, ethnicity, and chronic health conditions. The stronger protective effect observed in non-current smokers highlights the potential role of lifestyle factors in modulating this relationship. The findings suggest that higher a WWI value may act as a protective factor against dementia in British aging populations, underscoring its potential utility in dementia prevention and management strategies.

In most prior investigations, BMI has been the conventional metric for defining obesity, calculating an individual’s weight relative to height [21]. However, the relationship between BMI and dementia is multifaceted. Existing evidence shows that middle-aged individuals with obesity face a 2–3-fold increased risk of developing dementia later in life compared to their non-obese counterparts [39]. Paradoxically, other research indicates that higher BMI or fat mass in later life may be protective against dementia. Anjum et al. found that an increased BMI in old age was linked to a lower probability of suffering from dementia [15]. Wang et al. further demonstrated L-shaped relationships between total and regional fat mass ratios and dementia risk in individuals aged 60 years or older, independent of overall obesity [16]. Mooldijk et al. also observed that greater total and regional fat mass reduced dementia risk [17]. The results obtained by Qizilbash et al. conflict with the assumption that middle-aged obesity contributes to a higher risk of dementia in old age [18]. Natale et al. discovered that there exists a negative relationship whereby obesity is linked to lower rates of dementia and dementia-related mortality [19]. Additionally, research indicates that higher BMIs in old age are correlated with better cognitive function and lower mortality rates [20]. Despite its widespread use, BMI has significant limitations as a measure of adiposity [23,40]. Crucially, BMI fails to distinguish between fat mass and lean muscle mass, often misclassifying muscular individuals as overweight or obese [22]. It also provides no information about fat distribution [23]. The metric’s reliance solely on height and weight further renders it insensitive to variations in body composition across different age groups and ethnic populations with varying adiposity patterns at identical BMI values [23]. These fundamental shortcomings limit BMI’s utility in assessing obesity-related health risks, including dementia.

The WWI represents a novel anthropometric approach to assess central adiposity, calculated through the ratio of waist circumference to the square root of body weight [23]. This composite measure uniquely captures body composition characteristics by simultaneously accounting for abdominal obesity and overall body size [25]. Unlike traditional indices, the WWI demonstrates discriminative capacity in evaluating adiposity patterns across varying BMI classifications [25]. Its mathematical formulation provides enhanced insight into fat distribution profiles that may have distinct metabolic implications. These factors make the WWI a more reliable indicator for examining the impact of central obesity on health in older adults. Our research demonstrates that elevated WWI levels are linked to a lower likelihood of dementia among older individuals. This inverse relationship persisted whether the WWI was analyzed continuously or by quartiles, suggesting that higher WWI may serve as a protective factor against dementia and could inform prevention strategies for aging populations.

One of the key findings of this paper is that our data show significantly lower WWI levels in British older adults, with a mean value of 8.05, compared to their Chinese counterparts (mean = 11.1) and American counterparts (first quartile [Q1] = 9.02; fourth quartile [Q4] = 14.79) [33,35]. This marked discrepancy suggests that WWI distributions may differ across populations, potentially reflecting variations in body composition, especially central obesity, which are driven by regional and ethnic factors. The pronounced divergence in absolute values may underlie the inconsistent dementia associations observed (positive in Chinese cohorts vs. negative in UK data) [34]. These findings underscore the need to establish population-specific WWI reference ranges to ensure accurate interpretation of its health associations. Clarifying these population-level differences is critical for improving the generalizability of the WWI as a predictive tool for dementia risk in diverse aging populations and refining dementia prevention strategies.

The counterintuitive finding of a lower dementia risk with higher WWI among elderly British adults may be explained by multiple mechanisms. First, the “obesity paradox” could be at play, where greater energy reserves in individuals with a higher WWI buffer protect against neurodegenerative catabolic processes, delaying dementia onset [41]. Second, lipid-related pathways may offer protection; triglycerides associated with elevated WWI could enhance lipoprotein metabolism, potentially aiding in amyloid-beta plaque clearance [42]. Finally, the adipokine profile of adipose tissue in these individuals might tilt towards neuroprotection, with substances like adiponectin improving insulin sensitivity, reducing oxidative stress, and promoting neuronal survival [43].

This prospective cohort study represents a significant advancement by offering the first longitudinal evidence of the association between the WWI and dementia among older adults in the UK. Unlike cross-sectional studies, which can only establish associations at a single point in time and cannot determine the direction of causality, the longitudinal design of our study allows us to establish temporal precedence. By measuring the WWI objectively prior to the diagnosis of dementia, we can infer that changes in the WWI may precede the onset of dementia, providing crucial evidence for a potential causal relationship.

Nevertheless, our study is subject to several limitations. First, although we adjusted for multiple covariates, residual confounding may still have occurred due to unmeasured factors. This is because certain variables, which could potentially influence the study results, were not included in our initial data request from the UK Biobank. For example, the Townsend deprivation index and detailed physical activity metrics like metabolic equivalent of task minutes per week might be relevant. Incorporating these variables in future research could help reduce confounding and strengthen the reliability of the findings. Second, in addition to the significant interaction found in the smoking status subgroup analysis, we also conducted subgroup analyses for sex, smoking status, alcohol intake, education level, ethnicity, and chronic health conditions. However, the results of these subgroups did not show statistical significance. Several methodological constraints likely contributed to these findings. Smaller subgroup sample sizes compromised statistical power, while uneven case distribution across demographic and health-related categories may have obscured true associations. Unaccounted confounding factors could also have confounded results. Notably, although most interaction terms lacked statistical significance, the magnitude of certain coefficients hints at latent associations. These suggest that with increased sample sizes or refined analytical strategies, meaningful interactions may yet be revealed. Such potential for undetected relationships underscores the need for caution when interpreting our current results and generalizing findings across diverse subgroups. Future studies should prioritize larger, more balanced samples and comprehensive control of confounding to better elucidate subgroup-specific associations. Third, the mechanisms underlying the association between WWI and dementia, along with its public health implications, remain unclear. Our study identified an association, yet the biological pathways involved and the potential impact on public health policies for prevention and intervention have not been explored. Further research with mechanistic and population-based studies is needed to address these gaps.

## 5. Conclusions

Our study suggested negative associations between WWI and dementia risk among older adults in the UK. To our knowledge, this cohort study is the first to explore the association between the WWI and dementia risk in British aging populations. These findings imply that the WWI has the potential to predict dementia risk in older adults, underscoring its importance for dementia prevention strategies.

## Figures and Tables

**Table 1:** Basic characteristics of participants by WWI.

Characteristics	Overall	WWI			
		Q1 (<7.31)	Q2 (7.31–7.99)	Q3 (7.99–8.72)	Q4 (>8.72)
Count	155,683	38,934	38,910	38,918	38,921
Age at base (mean (SD))	63.23 (2.36)	63.30 (2.38)	63.28 (2.37)	63.27 (2.36)	63.07 (2.32)
Sex = 1 (%)	74,369 (47.8)	6275 (16.1)	15,436 (39.7)	23,609 (60.7)	29,049 (74.6)
BMI (mean (SD))	27.57 (4.46)	23.77 (2.64)	26.33 (2.74)	28.22 (3.05)	31.97 (4.48)
WWI_quantile (%)					
Q1	38,934 (25.0)	38,934 (100.0)	0 (0.0)	0 (0.0)	0 (0.0)
Q2	38,910 (25.0)	0 (0.0)	38,910 (100.0)	0 (0.0)	0 (0.0)
Q3	38,918 (25.0)	0 (0.0)	0 (0.0)	38,918 (100.0)	0 (0.0)
Q4	38,921 (25.0)	0 (0.0)	0 (0.0)	0 (0.0)	38,921 (100.0)
Ethnicity (%)					
White	145,821 (93.7)	35,847 (92.1)	36,431 (93.6)	36,658 (94.2)	36,885 (94.8)
Asian	3927 (2.5)	1212 (3.1)	974 (2.5)	915 (2.4)	826 (2.1)
Black	473 (0.3)	208 (0.5)	131 (0.3)	77 (0.2)	57 (0.1)
Mixed	4603 (3.0)	1257 (3.2)	1184 (3.0)	1118 (2.9)	1044 (2.7)
Other	859 (0.6)	410 (1.1)	190 (0.5)	150 (0.4)	109 (0.3)
Chronic condition (%)					
Heart attack	6308 (4.1)	839 (2.2)	1272 (3.3)	1723 (4.4)	2474 (6.4)
Angina	6628 (4.3)	1136 (2.9)	1464 (3.8)	1829 (4.7)	2199 (5.6)
Stroke	3085 (2.0)	627 (1.6)	711 (1.8)	817 (2.1)	930 (2.4)
High blood pressure	50,143 (32.2)	10,093 (25.9)	11,330 (29.1)	12,996 (33.4)	15,724 (40.4)
Other	89,519 (57.5)	26,239 (67.4)	24,133 (62.0)	21,553 (55.4)	17,594 (45.2)
College = 1 (%)	38,144 (24.5)	9222 (23.7)	9577 (24.6)	9910 (25.5)	9435 (24.2)
Ever drinker = 1 (%)	147,845 (95.0)	36,317 (93.3)	36,837 (94.7)	37,294 (95.8)	37,397 (96.1)
Current smoker = 1 (%)	12,402 (8.0)	3751 (9.6)	2936 (7.5)	2853 (7.3)	2862 (7.4)
Outcome = 1 (%)	3211 (2.1)	894 (2.3)	819 (2.1)	750 (1.9)	748 (1.9)

**Table 2:** Association between WWI and dementia.

Exposure	Model 1		Model 2		Model 3	
	OR (95%CI)	*p* Value	OR (95%CI)	*p* Value	OR (95%CI)	*p* Value
Weight-adjusted waist index (continuous)						
	0.95 (0.92, 0.99)	<0.01	0.92 (0.88, 0.95)	<0.0001	0.76 (0.71, 0.81)	<0.0001
Weight-adjusted waist index (quartile)						
Quartile 1	reference		reference		reference	
Quartile 2	0.91 (0.83, 1.01)	0.064	0.85 (0.77, 0.94)	<0.01	0.77 (0.69, 0.85)	<0.0001
Quartile 3	0.85 (0.76, 0.92)	<0.001	0.75 (0.67, 0.83)	<0.0001	0.61 (0.54, 0.69)	<0.0001
Quartile 4	0.87 (0.79, 0.96)	<0.01	0.77 (0.69, 0.85)	<0.0001	0.53 (0.46, 0.62)	<0.0001
*p* for trend	<0.0001		<0.0001		<0.0001	

**Table 3:** Subgroup analysis of association between WWI and dementia.

Subgroup	Case/Total	OR (95%CI)	*p* for Interaction
Sex			0.1680
Male	1549/81,314	0.75 (0.68, 0.82)	
Female	1662/74,369	0.76 (0.70, 0.83)	
Educational level			0.6886
College or university degree	611/38,144	0.71 (0.61, 0.82)	
No college or university degree	2600/117,539	0.77 (0.72, 0.83)	
Ethnicity			0.9210
White	2980/145,821	0.76 (0.71, 0.81)	
Asian	71/3927	0.77 (0.51, 1.15)	
Black	15/473	1.08 (0.44, 2.65)	
Mixed	121/4603	0.74 (0.54, 1.02)	
Other	24/859	0.45 (0.21, 0.96)	
Chronic condition			0.7459
Heart attack	217/6308	0.73 (0.58, 0.92)	
Angina	259/6628	0.94 (0.76, 1.17)	
Stroke	112/3085	0.69 (0.49, 0.96)	
High blood pressure	1105/50,143	0.71 (0.64, 0.79)	
Other	1518/89,519	0.79 (0.72, 0.86)	
Smoking status			0.0011
Current	293/12,402	0.72 (0.58, 0.89)	
Ever or never	2918/143,281	0.74 (0.69, 0.79)	
Alcohol use			0.3502
Ever or current	2961/147,845	0.77 (0.72, 0.82)	
Never	250/7838	0.69 (0.55, 0.86)	

## Data Availability

Restrictions apply to the availability of these data. Data were obtained from the UK Biobank and are available at https://www.ukbiobank.ac.uk/ with the permission of the UK Biobank.

## Abbreviations

The following abbreviations are used in this manuscript:

WWI	Weight-adjusted waist index
BMI	Body mass index
UK	United Kingdom
WC	Waist circumference
SD	Standard deviation
OR	Odds ratio
CI	Confidence interval
